# Supplementary figures and images for: Influence of the maternal high-intensity-interval-training on the cardiac Sirt6 and lipid profile of the adult male offspring in rats
Source: PLoS One. 2020 Aug 3;15(8):e0237148. doi: 10.1371/journal.pone.0237148 (PMC7398538; doi:10.1371/journal.pone.0237148)

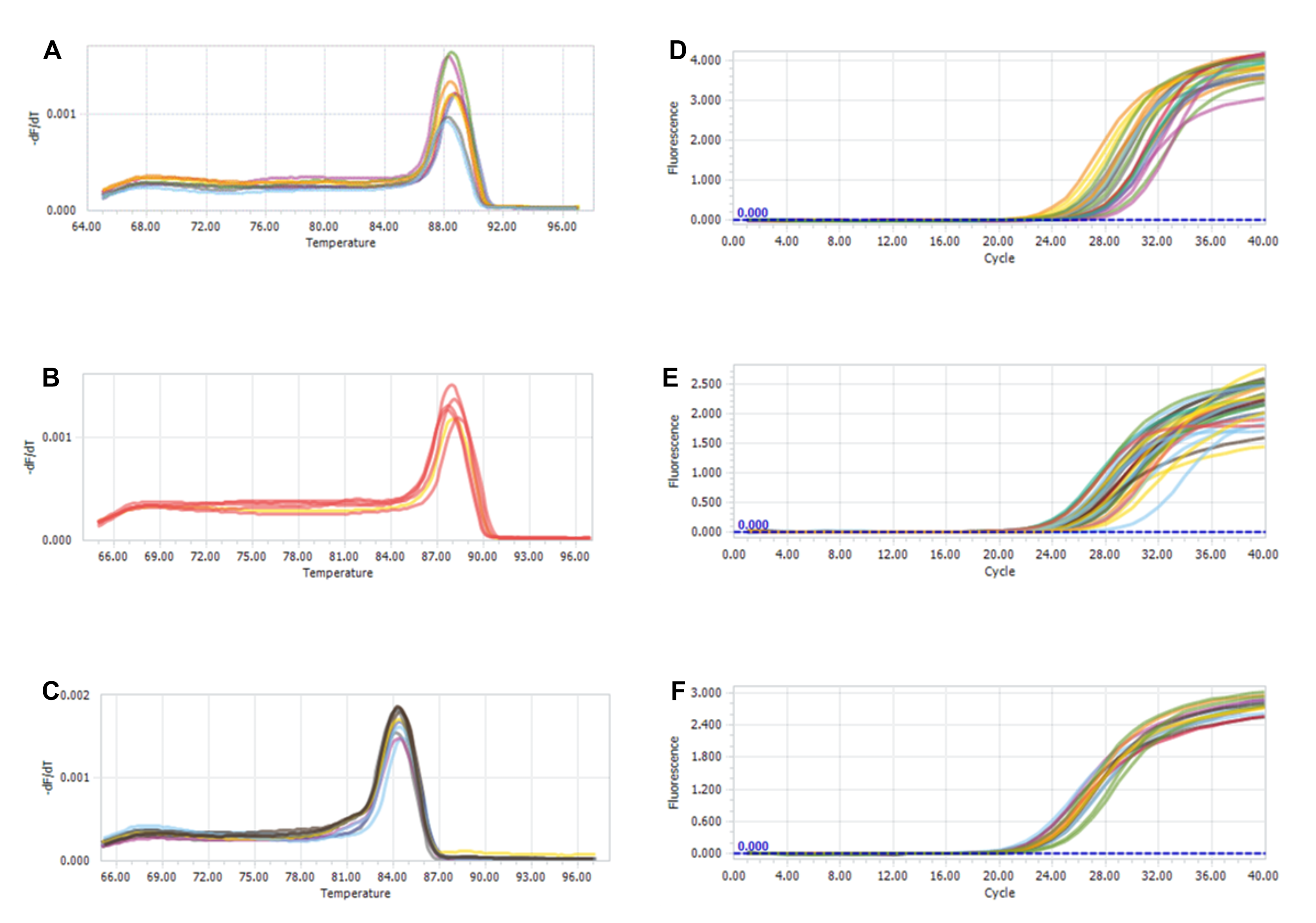

Supplement: S1 Fig — A) melting cure of sirt6, B) melting cure of igf2, C) melting cure of b-actin, D) Amplification plots of sirt6, E) Amplification plots of igf2 and F) Amplification plots of b-actin. (TIF) [file pone.0237148.s001.tif]
