# Supplementary material for: Influence of the maternal high-intensity-interval-training on the cardiac Sirt6 and lipid profile of the adult male offspring in rats
Source: PLoS One. 2020 Aug 3;15(8):e0237148. doi: 10.1371/journal.pone.0237148 (PMC7398538; doi:10.1371/journal.pone.0237148)
Supplement: Attachment — Submitted filename: Response to Reviewers.docx [file pone.0237148.s008.docx]

#### **Dear Christopher Torrens**

#### **Academic Editor**

#### **PLOS ONE**

The authors would like to Thank you for giving me the opportunity to submit a revised draft of our manuscript titled [*Influence of the maternal high-intensity-interval-training on the cardiac Sirt6 and lipid profile of the adult male offspring in rats, No. PONE-D-19-33225*] to *[****PLOS ONE****]*. We appreciate the time and effort that you and the reviewers have dedicated to providing your feedback on our manuscript. We have been able to incorporate changes to reflect most of the suggestions provided by the reviewers. We have highlighted the changes within the manuscript. We hope that this new version of the manuscript is satisfactory for the presentation and we are thankful once again to the reviewers for their useful comments.

Sincerely yours

**Neda Khaledi (Ph. D)**

Corresponding Author

**Academic editor' comments:**

We truly appreciate the Editor’s suggestion, and we recognize the benefits improving the quality of the manuscript.

**Point 1:** Is the birthweight the mean per litter, which in turn is meaned for the group?

Looking at this table again, I don't undertstand the sex distribution values given. It seems to correspond to the number of offspring but that value is presented as a mean +/- SEM. For example the number of pups in the BP group is given as 5 +/- 1.71, which suggests quite a large variation in pups number, if so what does it mean that the split was 3:2 M:F?

**Response:** Yes, this birthweight was reported based on the mean of all litter per group for example in the BP group is given as 6.020 ± 0.25, which this value is obtained from the meaning of pups’s birth weight of 6 mothers.

We agree with your suggestion about sex distribution and we have added SEM for it.

**Point 2:** Line 219: Subsection on offspring physical activity. The title here says "Maternal HIIT...could improve physical activity...". This is not really what you measured though and this becomes ambiguous. Your data show they could run longer and faster but this is not the same as being more active, which you acknowledge because the title of the section says "could improve". **Response:**  In accordance with your suggestion, we have revised this section.

**Point 3:** Table 5 needs standardised the decimal points. The raw mass is given to three decimal places but the error is only to two, while the percentage is give to two with the error given to three places. The two measure have difference accuracies and don't need to be standardised as such, but the error for each measurement should be in the same format as the measurement it is taken.

**Response:** You have raised an important point. It was corrected.

**Point 4:** Line 242 "Beneficial effects" This is not the you found result. The result is a change in sirt6 and IGF-2 mRNA expression in response to maternal HIIT protocols. Any interpretation of what this means, be it good or bad belongs in the discussion not the results.

**Response:** Thank you for a valuable point. It was corrected.

**Reviewers' comments:**
**Reviewer #1**: Manuscript Number: **PONE-D-19-33225**

We are grateful to the reviewer 1 and 2 for their insightful comments on this article.

**Point 1**: It should be made clear that “Maternal HIIT prior to and during pregnancy have beneficial effects on the heart of the next generation” are referring to the offspring – as written, it is hard to determine if the authors mean the offspring (F1) or the next generation (F2).

Response: Thank you for pointing this out. It was changed to offspring.

**Point 2**: Line 181: Should be “vena cava” instead of Veno Cova

Response: This mistake was corrected. Thank you.

**Reviewers' comments:**
**Reviewer #2**: Manuscript Number: **PONE-D-19-33225**

**Point 1:** The authors did not perform most of the experiments I proposed in my revision. They conclude that maternal exercise may have effects on cardiovascular health, yet they fail to do studies of cardiac function.

Response: Although faced with limitation just as you said, we believe that additional experiments including direct measurements of cardiac function are very useful and will strengthen our work, but unfortunately due to the lack of funding time we are unable to investigate further at this time. accurate assessment of cardiac function, which is one of the limitations of our research, need to be known in further work and this limitation was added in the manuscript (line 299). Indeed, in accordance with your suggestion we have removed the term of cardiovascular health and have revised the manuscript.
